# Implementation and early results of same-day hip and knee replacement in a public hospital without prior same-day surgical experience, a feasibility study

**DOI:** 10.1007/s00402-026-06468-0

**Published:** 2026-08-20

**Authors:** Christoffer C. Jørgensen, Thor-Magnus Sveen, Brita Johansen, Marlene Sander, Christian Steen-Hansen, Mikkel S. Sundstrup, Christian Rothe, Kai HW. Lange

**Affiliations:** 1 Department of Clinical Medicine, University of Copenhagen, Copenhagen, Denmark; 2 Department of Anaesthesia, Nordsjællands Hospital, Hillerød, Denmark; 3 Department of Orthopedic Surgery, Nordsjællands Hospital, Hillerød, Denmark; 4 The Centre for Fast-Track Hip and Knee Replacement, Copenhagen, Denmark

**Keywords:** Arthroplasty, Hip Replacement, Knee Replacement, Same-day surgery, Ambulatory surgery, Outpatient, Feasability, Day surgery

## Abstract

**Background:**

Hip and knee replacements are increasingly performed as same-day surgery (SDS). However, the feasibility of SDS pathways in departments without prior experience is unknown. We report on the implementation process, feasibility and early results of implementing a SDS protocol in a department without prior experience with same-day hip and knee replacement.

**Methods:**

The study design was based on Bowens feasibility study framework on implementation processes. Data were collected prospectively through patient reported questionnaires and medical records during, and from the local Department of Digitalisation and Analytics after implementation. Outcome data included discharge on day of surgery, 30-days primary-care contacts, 90-days readmissions and patient satisfaction.

**Results:**

Implementation was initiated as a dedicated fellowship from September 2023 to October 2024. Patients were admitted and discharged through a dedicated day-surgical department with transfer to the regular ward in case of admission. The first SDS patient underwent surgery the 4th of September 2023. By October 2024, 150 of 713 operated patients (21.0% CI:18.2–24.2) had SDS. Of these, same-day discharge was achieved in 125 (83.3% CI:76.6–88.5) patients of which 6 (4.0% CI:0.9–7.1) were readmitted within 90-days. From October 2024 to October 2025, 178 of 800 patients were planned for SDS (22.3% CI:19.5–25.3), of which 131 (73.6% CI: 66.7–80.1) achieved same-day discharge despite less restrictive inclusion criteria.

**Conclusion:**

Implementation of a protocolled SDS framework was feasible within 1 year despite no prior SDS experience. SDS utilisation and success remained stable the 12 following months, confirming adaptation into standard of care.

## Introduction

Total Hip (THR), total knee (TKR) and unicompartmental knee replacement (UKR) are common procedures which are only expected to further increase in the coming years [7]. At the same time public health-care systems are under an increasing strain due to limitations in financial and personnel resources which pose a challenge for many developed countries. Fast-track or enhanced recovery protocols have continuously improved postoperative convalescence after these procedures, resulting in vastly reduced length of hospital stay (LOS) and without increase in readmissions [20]. Given these above factors the interest in performing hip and knee replacements as same-day surgery (SDS) within public health-care systems has vastly increased in recent years [15]. In 2023, the Danish Center for Fast-track Hip and Knee Replacement collaboration (FHKR) consisting of eight high-volume departments, published a study protocol describing a clinical setup and study aims for SDS hip and knee replacement [19]. This was followed by a paper describing the stages of implementation across the collaborating departments with 95% of SDS eligible patients being included in an SDS-pathway and a SDS success rate of 21% of all patients [6]. However, although five of the eight departments in the FHKR did not have SDS experience prior to implementation, all departments had a close shared scientific and clinical focus. Thus, the feasibility of applying the protocol to a department outside of the collaboration and without the same focus and networking capability is uncertain. This may be problematic as lack of data on generalizability and how to handle issues regarding leadership, resource- and integration in clinical practice may pose substantial barriers for implementing hip and knee replacement as SDS [17]. In 2022 following the COVID-19 pandemic, the Hospital of Northern Zealand faced an imminent shortage of staff in the orthopaedic wards. This led to an increasing number of elective surgeries, including hip and knee replacement, being cancelled and often outsourced to the private sector. To reduce the number of cancellations, it was decided to establish a SDS pathway within the department of ambulatory surgery. Here we report on the feasibility of implementation, 90-days clinical and patient-related outcomes and sustainability of the SDS pathway 12 months after completion of the implementation process.

## Methods

Ethical approval was not needed according to Danish legislation. However, all patients provided informed consent for review of medical records and receiving questionnaires, but with an opt-out possibility for questionnaires. Permission to collect and store data was acquired from the Capital Region legal department j.nr. 2,400,532. The study was designed based on the Bowen framework for feasibility studies [3], which focuses on the following eight dimensions: Acceptability, Demand, Implementation, Practicality, Adaptation, Integration, Expansion, Limited-efficacy testing (Table 1). Apart from funding of the implementation fellowship from the hospital administration, no additional funding was acquired.

**Table 1:** Study questions and outcomes of interest according to Bowens acceptability framework for feasibility studies

Area of focus	Question asked	Outcomes of interest
Acceptability	Will patients be willing to participate in the SDS pathway? Are the patients satisfied?	Number of eligible patients having SDS surgery Follow-up on patient satisfaction through questionnaires
Demand	Is there a need for a SDS pathway?	Estimated number of avoided cancellations or referrals to the private sector
Implementation	Will SDS capacity be utilized? Will the patients be discharged on day of surgery?	Proportion of patients with planned SDS procedures Fraction of planned SDS patients with successful same-day discharge
Practicality	Challenges regarding capacity for postoperative x-rays. Limitations regarding opening hours at the department of ambulatory surgery	Evaluation of resource use due to postoperative control x-rays in SDS-patients. Construction of contingency plans for patients unable to discharge before closing hours
Adaption	Need for revision of the original to allow for local adjustments	Number of major changes needed
Integration	How does a SDS-pathway synergize with the in-patient pathway?	Assurance that resources are not simply removed from the in-patient to the SDS-pathway
Expansion	To what extent can the SDS-pathway be expanded outside of the initial clinical and scientific framework??	Estimation of expected inclusion and success rates in another setting
Limited Efficacy	Is it possible to assure a full integration into clinical practice, after the end of the implementation period?	Additional data on utilization and success rates 10 months after the end of the implementation period

*SDS* same-day surgery

### Data collection

Data on clinical and patient-related outcomes was collected using a mix of prospective and retrospective information based on patient-reported questionnaires and follow-up through electronical health-care records, largely like the data collected by the FHKR. Data included information on SDS eligibility, choice of anaesthesia, reasons for admission and 90-days follow-up through review of electronical medical records. All patients accepting to complete questionnaires were asked about whether they had contacted the healthcare system, including their general practitioner and the emergency department within the initial 30 postoperative days (POD). Patients were also asked about their satisfaction with the SDS pathway using a 10-point Likert-Scale with 1 being “very dissatisfied” and 10 being “very satisfied”, and whether they would repeat or recommend others to having arthroplasty within the SDS pathway [4]. Follow-up on sustainability of the implementation of the SDS pathway into clinical practice was done using administrative quality-improvement data from October 2024 to October 2025 through the local Department of Digitalisation and Analytics. These data included type of procedure, age and ASA-status, time spent in hospital and whether admitted in the SDS pathway or through the regular ward.

All data collected during the implementation process were stored using Research Electronic DataCAPture software [12].

### Statistics and power considerations

As this is a feasibility study on a predefined implementation period, no pre-study power analysis was done. For follow-up, we chose to include a convenience sample during the 12 months following the end of the implementation period. Numbers are reported as medians with interquartile ranges and proportions as numbers with percentages including 95% confidence intervals (CI) based on Wilson score interval. No level of significance was defined as statistical comparisons were unmeaningful given the continued refinement of the SDS pathway. Data analysis was conducted using SPSS v. 29.0.1.0 (IBM Cooperation Armonk N.Y. U.S.A.) and www.statskingdom for calculating CI for proportions.

### Setting

The Hospital of Northern Zealand includes a dedicated arthroplasty section performing about 700 annual procedures. A general adherence to fast-track principles including multimodal analgesia, early mobilization and discharge to own home with a median LOS of 1 day, was already standard of care at time of SDS implementation. Patients were preferably admitted and discharged from a dedicated elective surgical ward.

Neither the department of orthopaedics or the department of anaesthesia had prior clinical experience with SDS for hip or knee replacement and the clinical pathway deviated from the protocol of the Foundation Centre for Fast-track Hip and Knee Replacement with regards to the following:

- Routine scheduled morphine administration.
- No routine use of non-steriod anti-inflammatory drugs (NSAID).
- Routine adductor blockade after total knee replacement (TKR).

Furthermore, utilization of UKR was established during implementation period.

### The SDS-pathway

Patients having planned SDS procedures were admitted directly to the Department of Ambulatory Surgery (DAS) and scheduled as either the first or second procedure of the day. The nurses at the DAS were trained as both operating and postoperative anaesthesia care unit nurses, but only one had prior routine with hip and knee replacement prior to implementation. This person was allocated with responsibility for educating the remaining nursing staff together with the physiotherapists, and for local adaption of the postoperative analgesic regimen. Mobilization was initiated as early as possible, and every patient was evaluated and received instructions by a physiotherapist prior to discharge. The DAS was staffed until seven PM, with patients not being able to be discharged prior to six o’clock being transferred to the orthopaedic ward. Postoperative X-rays were taken after discharge prior to the patient leaving the hospital and public rehabilitation after discharge was not routinely offered. During the implementation period all patients received a phone-call by a DAS nurse the day after surgery and electronic contact to the orthopaedic ambulatory nurses was possible through the Emento App (Emento A/S, Aarhus, Denmark) as well as by telephone.

### Anaesthesia and surgery

Initially general total intravenous anaesthesia (TIVA) was standard, but later spinal anaesthesia became an option. The choice of general anaesthesia was done to avoid prolonged motor blockade which could delay mobilization [5]. TIVA was done using propofol and remifentanil with optional rocuronium 0.6 mg/kg in case of need for intubation. Postoperative analgesia initially included fentanyl 2–3 mikrog/kg but increased to 3–5 mikrog/kg based upon clinical feedback. In case of spinal anaesthesia, either isobaric or hyperbaric Bupivacaine 5 mg/ml was used, with initial doses of 2-2.5 ml but reduced to 1.4–1.8 ml in 2025. For THR standard incision was posterolateral with cementation in patients > 70 years. TKR and UKR patients received local infiltration analgesia with Ropivacaine by the surgeon.

As there was a lack of capacity for providing peripheral nerve-blocks at the DAS this was removed from standard treatment and only applied on an as needed basis. All patients received multimodal opioid-sparing analgesia, including 24 mg of dexamethasone, paracetamol and NSAIDs, but with routine preoperative administration of 5 mg morphine in TKR and use of both 4 mg Ondansetron and 50 mg Cyclizine as PONV prophylaxis.

### The implementation process

**Fig. 1:**
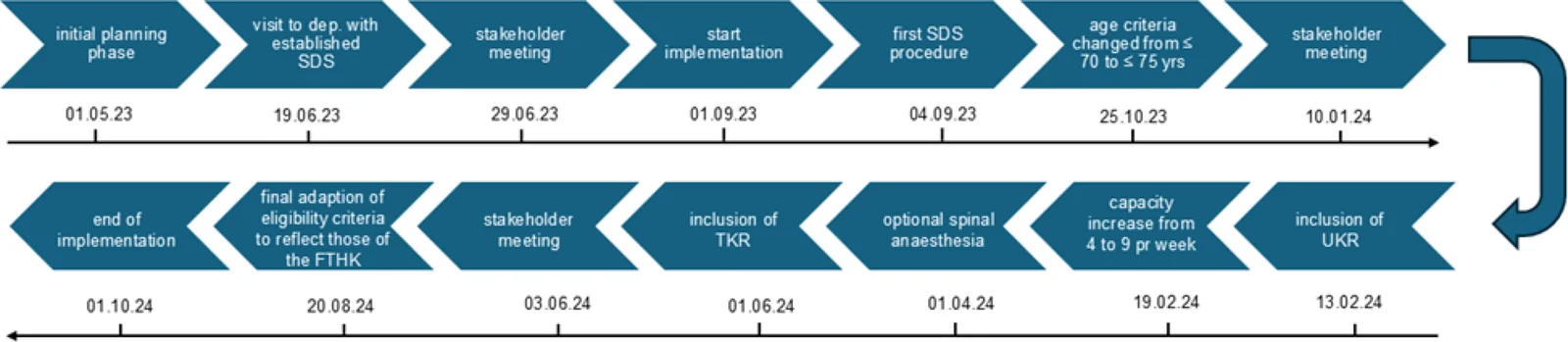
Timeline of the implementation process. *SDS* same-day surgery, *UKR* unicompartmental knee replacement, *TKR* total knee replacement, *FTHK* fast-track center for hip and knee replacement

The implementation was planned to be conducted as a dedicated 1-year part-time fellowship from September 2023 to October 2024. The fellowship programme at the Hospital of Northern Zealand is a local initiative and include sparring with other fellows and the project sponsor, as well as formal education focusing on healthcare improvement [18] based on the Plan-Do-Study-Act (PDSA) concept use of quality data for monitoring progress [2]. The fellowship was preceded by an initial visit to a department with established SDS and participating in the FHKR. This was followed by a multidisciplinary stakeholder meeting including nurses, physiotherapists, surgeons and anaesthetists. (Fig. 1).

During implementation local clinical pathways were revised, including use of routine NSAIDs and gastric ulcer prophylaxis, and morphine being changed to an “as needed basis”. To enhance the initial likelihood of same-day discharge and due to capacity limitations, eligibility criteria were restricted to only THR and age and BMI were set to maximum 70 years and < 35, respectively. General anaesthesia was preferred to avoid prolonged motor blockade delaying early mobilization. To facilitate discharge an agreement was made with the Department of Radiology allowing for X-ray of the prosthesis on day of surgery rather than next morning. Throughout the implementation, period teaching sessions, written checklists and other supportive documents for the DAS-nurses were developed and followed by qualitative interviews on usefulness and satisfaction [22]. Regular meetings were also held with the hospital board and heads of the departments of Orthopaedics and of Anaesthesiology.

The first patient underwent surgery on the 4th of September and throughout 2023 minor changes were done with regards to inclusion criteria and postoperative analgesia based upon continuous feedback from clinical staff (Fig. 1). From February 2024 SDS capacity was increased and included UKR, while TKR was included from June 2024. In August 2024 the eligibility criteria were finally adjusted to resemble those used by the FHKR [19].

## Results

A total of 713 THR, TKR or UKR procedures were conducted during the implementation period. Of these, 158 (22.2% CI: 19.1–25.2) were registered as eligible for SDS of which 150 (94.9% CI: 90.3–98.4) were included in the SDS pathway. Table 2 shows characteristics of the SDS patients. Median LOS was 0 (IQR: 0–0) with 1 patient staying 4 days due to a hip displacement. Discharge on day of surgery was achieved in 125 (83.3% CI:76.6–88.5) patients with the main reasons for not being discharged on day of surgery being PONV and insufficient mobilisation (Table 3). One of the admitted patients was not discharged until POD4 due to anterior hip displacement followed by revision surgery. The remaining 24 patients were all discharged on POD1. When contacting the patients the day after surgery 17 (13.6% CI: 8.7–20.6 of discharged patients) had issues related to postoperative care, of which 4 (3.2% CI: 1.2–7.9) required further healthcare contact. Causes of concerns were related to pain and mobilization in 8 cases, oozing from the wound or a feeling that something was wrong in the joint in 2 cases each, dizziness in four and constipation in five cases. Of those who had further healthcare contact, two had experienced syncope, one needed an additional wound suture, one had severe pain, one had persistent urinary retention, and one had a subluxation of the prosthesis.

**Table 2:** Patient characteristics

Characteristics *N* (%)	SDS patients during implementation *n*:150	SDS patients after implementation *n*:178
Age (median IQR)	65 (59–70)	67 (60–72)
Females	85 (56.7)	NA
ASA		
I	38 (25.3)	38 (21.3)
II	96 (64.0)	121 (68.0)
III	16 (10.7)	19 (10.7)
CSF (median IQR)	2 (1–3)	NA
Cohabiting	106 (77.9)	NA
Missing	14 (9.3)	
Smoking	24 (17.5)	NA
Missing	13 (8.7)	
Alcohol > 10 units/week	18 (13.1)	NA
Missing	13 (8.7)	
Analgesics for chronic pain	16 (11.3)	NA
Missing	8 (5.3)	
Treated pulmonary disease	5 (3.3)	NA
Missing	11 (7.3)	
Hypertension	52 (34.7)	NA
Missing	11 (7.3)	
Atrial fibrillation	5 (3.3)	NA
Missing	11 (7.3)	
Anticoagulant treatment	22 (14.7)	NA
Missing	11 (7.3)	
Rheumatic arthrosis	4 (2.7)	NA
Missing	11 (7.3)	
NIDDM	4 (2.7)	NA
Missing	11 (7.3)	
Psychiatric disorder	8 (5.3)	NA
Missing	11 (7.3)	
Procedure		
THR	119 (79.3)	117 (65.7)
TKR	6 (4.0)	32 (18.0)
UKR	25 (16.7)	29 (13.8)
Choice of anaesthesia		
General	111 (74.5)	NA
Spinal	38 (25.3)	
Missing	1 (0.7)	
First procedure of the day	133 (88.7)	NA
Missing	6 (4.0)	

*NA* not available, *ASA* American Society of Anesthesiology, *CSF* Clinical Frailty Score, *THR* total hip replacement, *TKR* total knee replacement, *UKR* unicompartmental knee replacement

**Table 3:** Causes of admission to hospital in SDS patients

Cause	*n* (%; CI)
Insufficient mobilisation	8 (5.3; 2.7–10.2)
Postoperative nausea and vomiting	9 (6.0; 3.2–11.0)
Pain	3 (2.0; 0.7–5.7)
Prolonged motor blockade	2 (1.3; 0.4–4.7)
Urinary retention	2 (1.3; 0.4–4.7)
Surgical complication	4 (2.7; 1.0-6.7)
Not informed about SDS	1 (0.7; 0.1–3.7)
No adult at home	1 (0.7; 0.1–3.7)

In total 25 of 150 patients were admitted. Total number of causes leading to admission was 30 due to some patients having > 1 issue

Of the 128 (75.9% CI: 69.9–81.2) of patients completing 30-days patient reported follow-up, 10 (7.8% CI: 4.3–13.8) reported admission to the emergency ward (Table 3). Contact to general practitioner was reported in 43 patients (33.6% CI 26.0-42.2), with the most common cause being pain and need for additional analgesic prescriptions. (Table 4) Finally, 6 patients (4.0% CI:1.8–8.5) had a readmission within the first 3 months occurring after median 66 days (IQR: 36–70), and 1 patient had a planned revision on POD27 due to a periprosthetic fracture which had likely occurred during the primary procedure.

**Table 4:** Patient reported causes of postoperative health-care contacts

Cause	30-days general practitioner *n*:43 (33.6%)	30-days emergency room *n*:10 (8.1%)
Insufficient mobilisation	3 (2.4% CI: 0.8–7.9)	0
Disproven DVT	3 (2.4% CI: 0.8–7.9)	2 (1.6% 0.4–5.7)
Swelling	2 (1.6% CI: 0.4–5.7)	3 (2.4% 0.8–7.9)
Hip displacement	1 (0.8% CI:0.1–4.5)	2 (1.6% 0.4–5.7)
Planned removal of sutures	5 (4.1% CI: 1.7–9.2)	0
Other wound complications	3 (2.4% 0.8–7.9)	1 (0.8% CI:0.1–4.5)
Pain and analgesic prescription renewal	11 (8.9% CI: 5.1–15.3)	0
Concerns about infection/Prosthetic malfunction	5 (4.1% CI: 1.7–9.2)	0
Other prescription renewal	2 (1.6% 0.4–5.7)	0
Fever	3 (2.4% 0.8–7.9)	1 (0.8% CI:0.1–4.5)
Urinary tract infection	1 (0.8% CI:0.1–4.5)	1 (0.8% CI:0.1–4.5)
Urinary retention	1 (0.8% CI:0.1–4.5)	0
Unrelated to surgery	2 (1.6% 0.4–5.7)	0
Unknown	1 (0.8% CI:0.1–4.5)	0

In total 123 (82%) of 150 patients completed the 30-day patient reported follow-up

*DVT* deep venous thromboembolism

Among the 113 (75.3% CI:67.9–81.5) patients reporting on satisfaction, 104 were discharged on day of surgery and median satisfaction was 9 (IQR: 8–10). Of 95 respondents discharged on day of surgery, 85 (89.5% CI: 81.7–94.2) would prefer having surgery within the SDS pathway in case of subsequent arthroplasty procedures. Finally, 83 of the 95 respondents (86.8% CI: 80.6–93.1) would recommend the SDS pathway to others.

### Sustainability and integration into clinical practice

During the initial 10 months following the implementation process 800 procedures were performed according to data from the Department of Digitalisation and Analytics. Of these 178 (22.3% CI: 19.5–25.3) were included in the SDS pathway (Table 2). Median LOS of SDS patients remained 0 days (IQR:0–1), and 131 (73.6% CI: 66.7–79.5) were discharged on day of surgery after a median of 8.2 h (IQR: 7.4-9.0). Of the 47 (26.4% CI:20.5-33-3) admitted patients, 41 went home the day after surgery and 6 had a LOS of ≥ 2 days. Of the patients who were not included in the SDS pathway, 103 (16.6% CI: 13.9–19.7) were also discharged on day of surgery, but from the regular ward, after a median of 8.8 h (IQR: 5.8–11.7).

## Discussion

This study demonstrates the feasibility of implementing a SDS pathway based upon a previously protocolled scientific clinical collaboration [19] in a department outside of the framework and without prior SDS experience. Furthermore, our study may inform future multi-centre studies on efficacy with regards to incorporating the SDS pathway into everyday clinical practice, also after the end of the implementation process. Finally, the high satisfaction rates and willingness to repeat having SDS in case of a second major joint replacement confirmed the acceptability of the SDS pathway amongst the patients. The results of our study need to be confirmed in other hospital settings, but may provide baseline estimates of what to expect in countries where SDS pathways are not yet widely implemented. To our knowledge there has only been one prior study investigating implementation and early outcomes of a “short-stay” protocol in hip and knee replacement from the U.K [14]. Importantly, their aim was an overall reduction in LOS from a median of 4 days and with about 15% of patients having SDS one year after implementation [14]. Thus, SDS remains rare in the U.K. with only about 1% of THR and 10% of UKR performed on an outpatient basis in 2022 [8]. 

One of the reasons for the speed of our implementation may be the financial support from the hospital board. This allowed dedicated personnel to ensure that the experiences from the clinical staff and patients were used for modifying the pathway accordingly. In example, we had initial issues with PONV after THR, as has also been found previously [5]. This led to inclusion of Cyclizin as preoperative prophylaxis which reduced the problem. Similarly, the initial preference for general anaesthesia was to limit problems regarding prolonged motor blockade after spinal anaesthesia [5, 16]. This did become a problem, but diminished considerably after the launch of a project to determine the most suitable doses of anaesthesia. Thus, continued development of the protocol may further reduce postoperative issues that may correctable by timely appropriate anaesthetic, nursing or physiotherpay interventions.

Prior to implementation we decided to adapt the proposed inclusion criteria to increase likelihood of same day discharge. This was done to increase the feeling of success among staff and to avoid straining resources in the regular ward, which was undermanned on nursing staff. Although eligibility criteria for SDS remain controversial [10, 19, 23], we decided to start by including THR patients only as these, in contrast to most literature [9], have been found more likely to achieve same-day discharge than TKR patients in FTHK data [5]. Although UKR patients are the most suitable SDS candidates [9], inclusion of UKR was postponed due to a lack of surgical expertise at the start of implementation. We also lowered the eligible age and BMI cut-offs with the aim of further increasing likelihood of success at the early stages of implementation [9]. Nevertheless, the success rate of about 75% is higher than the 52% reported by Danielsen et al. within the initial 6 months of implementation within the FTHK [6], and similar to the 60–70% in a subsequent report after implementation [5]. While the reason for the initial success rate may be related to stricter patient and procedure selection criteria, the inclusion criteria after implementation were similar to the FTHK and general criteria in the Capital Region. Despite this adjustment, we registered only a small drop in same day discharge rates. One possible explanation for this may be that, in contrast to in the FTHK, our setup is based upon discharge directly from the DAS. However, this approach pose challenges due to limited opening hours of the DAS, which in our setting necessitates discharge before 6 PM. Furthermore, it may influence SDS capacity which in our case is only to about 25%, considerably lower than in other studies reporting up to 85% [5]. Regarding readmissions, these were rare, especially within the initial 2–3 days, although 1 patient did have a primary surgical complication resulting in need for planned revision surgery. In contrast, about 35% contacted their general practitioner within the first month, often due to pain related issues. The need for healthcare contacts after discharge is not well investigated, but our data are somewhat higher than the 25% reported in a recent study [13]. Willingness to repeat SDS surgery has previously been found in 90% of patients [4], similar to our study. Importantly, after implementation, patients were no longer actively asked to choose SDS as it became a standard of care. This may be an important cultural change, as about 20% of patients are uncertain about having SDS surgery prior to the procedure, but with 30% of these having a positive opinion after surgery [11].

Our study has some limitations. Thus, data was prospectively acquired during but not after the implementation process, limiting analysis on postoperative outcomes and satisfaction. Furthermore, not all patients answered the questionnaires, introducing the risk of selection bias. Also, the questionaire on patient satisfaction was not validated, but chosen to reflect what was used in a previous Danish multicenter study to enable comparison with departments with well established same day discharge pathways [4]. Furthermore, the first author who headed the project, was part of the FTHK collaboration and had access to the FTHK data. However, the results of the FTHK are now available in the scientific litterature. Finally, although there was no prior experience with SDS hip and knee replacement at the Hospital of Northern Zealand, ERAS principles were widely implemented with a LOS of median 1 day, providing a solid foundation for implementing a SDS pathway [24]. Thus, whether our results can be directly applied outside of the Danish healthcare system or in departments with 3–4 days of hospitalisation despite ERAS protocols as often seen in other countries [1, 21] is uncertain. Strengths of our study include documentation on all aspects of implementation, the use of an evidence-based published protocol for basing the SDS framework and prospective datacollection enabling reasonable comparisons to recent SDS studies.

In conclusion, our study demonstrated the feasibility of implementing a SDS protocol for hip and knee arthroplasty in a department without prior SDS experience. Following the implementation process, transition into clinical practice was satisfactory without apparent safety issues, and high patient satisfaction.

## Data Availability

Data is not widely available due to Danish legislation. Access to anonymized data can be acquired from the authors upon request.
